# Surgical site infections caused by multi-drug resistant organisms: a case–control study in general surgery

**DOI:** 10.1007/s13304-022-01243-3

**Published:** 2022-03-19

**Authors:** Diego Foschi, Al’ona Yakushkina, Francesco Cammarata, Giulia Lamperti, Francesco Colombo, Sara Rimoldi, Spinello Antinori, Gianluca M. Sampietro

**Affiliations:** 1 Second Unit of General Surgery, S. Joseph Hospital, Multimedica IRCCS, Milan, Italy; 2 Division of General Surgery, “Luigi Sacco” University Hospital, ASST Fatebenefratelli-Sacco, Milan, Italy; 3 Unit of Clinical Microbiology, “Luigi Sacco” University Hospital, ASST Fatebenefratelli-Sacco, Milan, Italy; 4 Department of Infectious Diseases, “Luigi Sacco” University Hospital, ASST Fatebenefratelli-Sacco, Milan, Italy; 5 Division of General and HBP Surgery, Rho Memorial Hospital, ASST Rhodense, Corso Europa 250, 20017 Rho, Milano Italy

**Keywords:** Surgery, Complications, Surgical site infections, Multi-drug resistance, Risk factors, Cost of care

## Abstract

Multi-drug resistant organisms (MDR-Os) are emerging as a significant cause of surgical site infections (SSI), but clinical outcomes and risk factors associated to MDR-Os-SSI have been poorly investigated in general surgery. Aims were to investigate risk factors, clinical outcomes and costs of care of multi-drug resistant organisms (MDR-Os-SSI) in general surgery. From January 2018 to December 2019, all the consecutive, unselected patients affected by MDR-O SSI were prospectively evaluated. In the same period, patients with non-MDR-O SSI and without SSI, matched for clinical and surgical data were used as control groups. Risk factors for infection, clinical outcome, and costs of care were compared by univariate and multivariate analysis. Among 3494 patients operated on during the study period, 47 presented an MDR-O SSI. Two control groups of 47 patients with non-MDR-O SSI and without SSI were identified. MDR-Os SSI were caused by poly-microbial etiology, meanly related to Gram negative *Enterobacteriales*. MDR-Os-SSI were related to major postoperative complications. At univariate analysis, iterative surgery, open abdomen, intensive care, hospital stay, and use of aggressive and expensive therapies were associated to MDR-Os-SSI. At multivariate analysis, only iterative surgery and the need of total parenteral and immune-nutrition were significantly associated to MDR-Os-SSI. The extra-cost of MDR-Os-SSI treatment was 150% in comparison to uncomplicated patients. MDR-Os SSI seems to be associated with major postoperative complications and reoperative surgery, they are demanding in terms of clinical workload and costs of care, they are rare but increasing, and difficult to prevent with current strategies.

## Introduction

Healthcare associated infections (HAI) cause significant morbidity and mortality in subjects admitted to acute care hospitals [1–4]. The Center for Disease Control and Prevention (CDC) reported in the USA, between 2011 and 2014, 365,490 HAI caused by central line-associated bloodstream infections (CLABSI) [23.5%], catheter associated urinary tract infections (CAUTI) [37.8%], ventilator-associated pneumonia (VAP) [2.2%], and surgical site infections (SSI) [36.4%]. About 50% of the SSI were related to abdominal surgery, with prevalence depending on the type of operation [1]. The European Center for Disease Control and Prevention (ECDC) reported the prevalence of HAI in Europe as high as 3.4%, with 4.2 million HAIs in 2013 [2]. Even though HAI decreased in the last decade, a shift from naïve bacteria to drug-resistant organisms has been observed, resulting in a worse prognosis for patients and increased costs for the Health Systems [1, 3, 5, 6]. *Staphylococcus aureus* and *Escherichia coli* are the most frequently isolated bacteria from SSI culture, with a resistance rate ranging from 42.7 to 44.7% for *S. aureus,* and from 13.3 to 15.3% for *E. coli* [1].

Prior hospitalization, previous antibiotic treatments and preoperative infections are risk factors causing antibiotic resistance [7–9]. As proposed by the ECDC, bacteria should be classified as multi-drug resistant (MDR-Os), extended-drug resistant (XDR-Os), and pan-drug resistant organisms (PDR-Os) when they have developed resistance to one or two, three or more, or to all known antibiotics respectively [10]. MDR-Os infection has been studied in patients undergoing transplantation, oncologic, and emergency surgery [11–21], but if resistance increases morbidity and mortality in the general population [6, 22], it is not clear if it plays the same role in surgical populations [12, 17–19].

The aim of the present study was to investigate, in surgical patients affected by SSI, the risk factors and the effects of MDR-Os infections, and to quantify the extra-costs for their treatment.

## Patients and methods

From January 2017 to December 2019, following the indication of the National health authorities’ program against antibiotics resistance (PNCAR) [23], a systematic microbiologic investigation of all patients affected by SSI was prospectively performed, to assess incidence, risk factors, impact on clinical course, and healthcare costs of MDR-Os related infections in surgical patients. SSI was defined as any infection occurred within 30 days after the operation and classified as superficial, deep or organ-space according to Culver [24]. Follow-up included an outpatient visit within 30 days after the hospital discharge. The study was submitted to and approved by the Ethic Committee of the L. Sacco Hospital (#00-28004), and an informed consent was obtained from all the patients. The study was conducted according to the ethical standards of the Declaration of Helsinki (2013 version) and reported according to the Strengthening the Reporting of Observational Studies in Epidemiology [STROBE] guidelines [25, 26].

### Study design

The aim of the study was to evaluate the effects of MDR-Os infection on the clinical course of patients undergoing general surgery. Primary endpoints were morbidity and mortality, classified according to Clavien–Dindo. Grade 1 and 2 were considered minor complications, grade 3–4 major complications, and grade 5 corresponds to the death of the patient [27]. Secondary endpoints were the identification of risk factors for MDR-Os infection and determination of the extra-costs caused by MDR-Os infections. Major complications were very frequent in patients affected by MDR-Os SSI (> 50%) and very rare in patients with non-MDR-Os SSI (< 10%) or without SSI (≈ 1%). From the nomogram of Feigl, with a 95% confidence limit and interval of 50% a number of observations = 35 in each group was considered sufficient for the analysis. Therefore, we decided to maintain the number of 47 observations and to include two control groups: one group of patients affected by non-MDROs SSI and a second of patients without SSI. Control groups were selected in the same time frame of MDR-Os patients, and standardized by age, male/female ratio, diagnosis, and surgical treatment.

The following preoperative risk factors were considered for the analysis: prior hospitalization within 12 months; prior antibiotic or immunosuppressive therapy ( Steroid, azathioprine, mercaptopurine or infliximab and analogs) within 3 months; preoperative hospital stay (days); body mass index (BMI [weight kg/height m^2^]) < 18.5 or > 30; weight loss > 5% in the last 3 months or > 10% without time limits; Type 2 diabetes mellitus (basal plasma glucose > 120 mg/dL and glycated hemoglobin > 6.5%); American Society of Anesthesiology (ASA) Physical Status > 2; blood values of total protein (g/L), albumin (g/L), transthyretin (TST) (mg/dL), C-reactive protein (mg/L), red blood cell count (RBC 10^12^/L), hemoglobin (g/L), white blood cell count (WBC 10^9^/L), and lymphocyte count (LC 10^9^/L). Peri-operative surgical data considered in the study were: indication for surgery; elective or emergency setting; open or laparoscopic approach; pre-existing infections; surgical wound classification (clean, clean-contaminated, contaminated, dirty-infected, according to Mangram [28]); duration of the surgery (min); abdominal drainage insertion; open abdomen treatment; and iterative surgery (number of reoperations). To evaluate the clinical course of the patients affected by MDR-Os, the following postoperative parameters were registered: duration of postoperative hospital stay (days); total hospital stay (days); surgical (SSI, dehiscence, fistula, hemorrhage, occlusion) and non-surgical (cardio-vascular and respiratory) complication rate; non-SSI complications rate (CLABSI, CAUTI and VAP cases were recorded if positive microbiological cultures were obtained); the type, posology and duration of the antibiotic treatment; admission to the Intensive Care Unit (ICU) and its duration (days); parenteral and/or enteral nutrition; blood transfusion; naso-gastric tube; central venous catheter; urinary catheter for more than 3 days. Evolution from infection to sepsis, septic shock and organ failure was recorded according to the Third International Consensus definitions for sepsis and septic shock (Sepsis-3), using the quick sequential organ failure assessment score (qSOFA) > 2 as threshold criteria [29].

### Microbiology

All the patients received broad-spectrum antibiotic prophylaxis except patients with infection at time of surgery who had empiric interval antibiotic therapy until cultures and antibiotic Minimum Inhibiting Concentration (MIC) were available. Patients with SSI or septic collection underwent culture of fluid sample, blood, urine, bronchoalveolar lavage and invasive devices when indicated. Semi-quantitative and qualitative cultures were plated in standard microbiological media and incubated for 48 h both aerobically and anaerobically. Antimicrobial susceptibility was determined by the disk diffusion technique. In selected cases, the MIC gradient strip test (Etest, bioMérieux, France) was performed following the European Committee on Antimicrobial Testing (EUCAST) recommendations [28]. The main categories of resistance were: methicillin resistant *Staphylococcus aureus* (MRSA), vancomycin-resistant *Enterococcus faecium* (VRE), extended spectrum beta-lactamase (ESBL)-producing *Escherichia coli* and *Klebsiella* species, *Pseudomonas aeruginosa* and *Acinetobacter *species resistant to third generation cephalosporins and carbapenems, Carbapenem resistant Enterobacteriales (CRE).

### Analysis of the MDR-Os SSI induced costs

To quantify the extra-cost of MDR-Os infections we considered the reimbursement obtained by the National Health System, based on the diagnosis-related group classification system (DRG), which calculates the diagnosis, the surgical procedure and the occurrence of complications [30]. The reimbursement is increased by a diary cost until a threshold length of stay is reached; thereafter the charge per diem is reduced. The cost of the antibiotic therapy was determined on the basis of the price established by the Italian national drug authority (AIFA) for each dose of the drug [31].

### Statistical analysis

Differences among groups were calculated using a two-tailed, Fischer’ exact test for categorical variables, and a Student *t* or Mann–Whitney *U* test where appropriate. Binary and multinomial logistic regression were also performed. To conduct the multivariate statistical analyses, biochemical continuous variables were transformed into categorical variables using laboratory reference cut-off values. The duration of TPN, INT, ICU stay, and hospitalization were not considered in the multivariate analysis. Preoperative stay was considered in case of more than one night spent in the hospital before the surgical intervention. Results are reported as OR with 95% confidence interval. Values of *p* < 0.05 were considered significant. Statistical analysis was performed using IBM SPSS Statistics v.25.

## Results

From January 2018 to December 2019, a total of 3494 operations were performed at the Second Unit of General Surgery of Luigi Sacco University Hospital. Abdominal procedures were 1970, and clean extra-abdominal procedures 1524. There were 472 cases of Inflammatory Bowel Disease (IBD), 355 gastro-intestinal cancer cases, 158 bariatric patients, 700 miscellaneous operations (mainly cholecystectomy and laparoscopic hernias), and 285 emergency surgeries. SSI rate was 4.43% (155 cases) and 47 patients presented an MDR-Os SSI, with an overall incidence of 1.34%. The highest rate was registered after emergency surgery (14/285 cases, 4.91%), when infection was present at the time of operation; IBD surgery had the second rank with 17/472 cases (3.64%), followed by oncologic surgery (10/355 cases, 2.81%), and bariatric surgery (3/158 cases, 1.8%). Miscellaneous operations had the lowest incidence of MDR-Os infection (3/700 cases, [0.42%]). There was a significant difference between clean and other surgeries (*p* < 0.00001), but not among the other abdominal procedures. MDR-Os patients had a mean age of 59.8 ± 17.4, a M/F ratio of 1.23, and the same surgical indications of the control groups, but there were more elective surgeries (72.3%), laparotomic approach (61.7%) and contaminated-dirty operations (59.5%). In Table 1 are reported the differences among groups in terms of surgical parameters. MDR-Os SS had more reoperation rate and open abdomen treatment, while patients without SSI had a significantly shorter operation time, more laparoscopic procedures and clean or clean-contaminated operations, less use of abdominal drains, and no iterative surgery or open abdomen.

**Table 1:** Surgical details

Operation related factors	MDR-O SSI (*N*: 47 [%])	Non MDR-O SSI (*N*: 47 [%])	NO SSI (*N*: 47 [%])
Elective/emergency	34/13	32/15	36/11
Laparotomic/laparoscopic	29/18	29/18	17/30*
Garner’s class 1/2/3/4	3/16/20/8	1/22/17/7	9/36/2/0**
Time (min)	222 ± 99	200 ± 93	148 ± 81*
Abdominal drain	46 (97.8%)	46(97.8%)	37(78.7%)**
Iterative surgery	20(42.5%)^§^	1(2.1%)	0
Open abdomen	5(10.6%)	0	0

^*^p < 0.05 vs MDR-O SSI. **p < 0.01 vs MDR-O SSI. ^§^*p* < 0.01 vs non-MDR-O SSI

Several clinical and biochemical findings resulted as risk factors associated to SSI (Table 2). In the multinomial logistic regression analysis, the only variable that reached statistical significance between MDR-O and non-MDR-O patients was transthyretin. MDR-O SSI patients were characterized by a significant presence of comorbidities, and transthyretin levels < 0.20 mg/dL in comparison to patients without SSI. The length of hospital stay in patients without SSI, with non-MDR, and with MDR-SSI was 10.19 ± 5.2, 18.3 ± 8.2, and 47.8 ± 42 days respectively.

**Table 2:** Clinical and biochemical risk factors for MDR-O SSI

	Univariate analysis			Multivariate analysis			
Risk factor	MDR-O SSI (*N*: 47 [%])	Non MDR-O SSI (*N*: 47 [%])	No SSI (*N*: 47 [%])	MDR vs. Non MDR OR (CI 95%)	Sig	MDR vs. No SSI 0OR (CI 95%)	Sig
Previous hospital admission	27 [57.4%]**	22[46.8]**	7 [14.8%]	2.11 (0.46; 9.68)	0.337	1.62 (0.23; 11.39)	0.627
Previous antibiotic therapy	15[31.9%]*	18 [38.3%]**	5 [10.6%]	0.24 (0.05; 2.54)	0.084	2.32 (0.25; 21.5)	0.459
Previous immunosuppression	6 [12.7%]	9 [19.1%]	3 [6.3%]	0.524 (0.108; 2.54)	0.422	2.33 (0.288; 18.8)	0.428
BMI (kg/m^2^)	25.2 ± 6.48	24.7 ± 5.2	25.6 ± 4	–	–	–	–
BMI < 18.5 and > 30	13 [27.6%]*	7 [14.8]	4 [8.5%]	1.42 (0.38; 5.28)	0.596	1.46 (0.246; 8.68)	0.676
DMT2	8 [17%]	9 [19.1%]	3 [6.3%]	0.655 (0.15; 2.91)	0.577	2.92 (0.385; 22.2)	0.3
Weight loss	26 [55.3%]**	20 [42.5%]*	9 [19.1%]	0.33 (0.07; 1.54)	0.157	0.031 (0.001; 1.25)	0.066
ASA score 3–4	22 [46.8%]*^,§^	11 [23.4%]	5 [10.6%]	2.52 (0.77; 8.24)	0.127	4.74 (1.02; 22.05)	0.047
Preoperative stay (days)	8 ± 10.5**^,§^	3.1 ± 4.3	2.6 ± 4.8	2.99 (0.83; 10.8)	0.094	1.19 (0.26; 5.4)	0.818
Hemoglobin (g/L)	12.2 ± 2.2	11.9 ± 2.5*	12.9 ± 2	0.287 (0.07; 1.54)	0.075	0.68 (0.138; 3.39)	0.644
RBC (× 10^12^/L)	4.44 ± 0.8	4.52 ± 1.4	4.6 ± 0.6	1.64 (0.44; 6.11)	0.46	2.86 (0.47; 17.32)	0.254
WBC(× 10^9^/L)	9.5 ± 4.9	9.4 ± 3.7	9.3 ± 4.7	1.33 (0.4; 4.42)	0.639	1.09 (0.24; 4.93)	0.904
Lymphocyte (× 10^9^/L)	1.78 ± 0.9	1.74 ± 0.3**	1.96 ± 0.40	–	–	–	–
CRP (g/L)	54.4 ± 84.8	55.7 ± 75.3	29.7 ± 64.5	1.81 (0.5; 6.53)	0.363	0.56 (0.13; 2.36)	0.429
Total proteins (g/L)	60.3 ± 7.9**^,§^	64.1 ± 6.6*	66.9 ± 5.6	1.02 (0.25; 4.08)	0.979	4.28 (0.68; 26.8)	0.12
Albumin (g/L)	30.7 ± 6.7**^,§^	33.6 ± 5.9**	36.6 ± 3.9	1.9 (0.29; 13.32)	0.499	1.09 (0.047; 25.5)	0.955
Transthyretin (g/L)	0.15 ± 0.06**^,§^	0.18 ± 0.05**	0.2 ± 0.03	9.54 (1.2; 75.8)	0.033	171 (4.49; 6522)	0.006

*p < 0.05 vs No SSI. **p < 0.01 vs No SSI. ^§^*p* < 0.05 vs non-MDR-O SSI. ^§§^*p* < 0.01 vs non-MDR-O SSI

In Table 3 are reported the univariate and the multivariate analysis of peri-operative complications. In the multivariate analysis, MDR-O SSI postoperative course was characterized by a higher reoperation rate and complications (Clavien–Dindo 3 and 4). An association between multi-drug resistance and multi-microbial infection was the most predictive factor of high-grade surgical complications. Furthermore, patients with MDR-O SSI had a higher rate of dehiscence and organ-space SSI. This increased the risk of further complications after surgery, such as reoperations, septic shock, and multi organ failure.

**Table 3:** Periioperative data

Postoperative course	MDR-O SSI (*N*: 47 [%])	Non MDR-O SSI (*N*: 47 [%])	Mult. analysis OR (CI 95%)	Sig
Minor complications C–D 0/I–II	0/19 [40.4%]	0/42 [89.3%]	5.373 (1.59; 18.13)	0.007
Major complications° C–D III–V	28 [59.5%]^§§,**^	5 [10.6%]		
General complications				
Cardiovascular	3 (6.8%)	0	–	0.084
Respiratory	11 (23.4%)^§^			
Surgical complications				
Hemorrhage	5 [10.6%]	4 [8.5%]	–	0.149
Dehiscence	16 [34%]^§^	3 [6.3%]		
Infectious complications				
Superficial SSI	20 [42.5%]	25 [53.1%]	–	0.052
Deep SSI	11 [23.4%]	19 [40.4%]		
Organ/Space SSI	16 [34%]^§^	3 [6.38%]		
Pneumonitis	5 [10.6%]	0	–	0.087
CLABSI	7 [14.8%]	5 [10.6%]		
CAUTI	10 [21.8%]	5 [10.6%]		
Septic evolution qSOFA > 2	47[100%]^§§^	17 [36.1%]	–	0.072
Septic shock	13 [27.6%]^§^	2 [4.2%]	–	0.373
Multi organ failure	6 [12.7%]^§^	0	–	0.270
Multi-microbic infection with C–D III–V	12 [25.5]^§^	1 [2.13%]	–	0.335
Reoperation rate	20 [42.5%]^§§^	1[2.1%]	12.2 (1.37; 109.2)	0.025
ICU admission	20 [42.5%]^§§^	5 [10.6%]	–	0.2
ICU stay (days)	12.8 ± 15.9^§^	1.2 ± 0.45		
Total postoperative course	38.1 ± 22^§§,**^	16.1 ± 7.3^*^		
Total hospital stay	47.8 ± 42^§§,**^	18.36 ± 8.2^*^		

*CLABSI* central line-associated bloodstream infections, *CAUTI* catheter associated urinary tract infection, *qSOFA* quick score for sepsis, *ICU* intensive care unit

*p < 0.05 vs No SSI. **p < 0.01 vs No SSI. ^§^*p* < 0.05 vs non-MDR-O SSI. ^§§^*p* < 0.01 vs non-MDR-O SSI

During the postoperative course, summarized in Table 4, MDR-Os infected patients needed blood transfusions and Mechanical Assisted Ventilation (MAV) more frequently than non-MDRO-SSI patients, and were the only to receive a tracheostomy. TPN use and duration was significantly higher in MDR-Os SSI patients in comparison to non-MDR-Os SSI and non-infected patients. Immuno-nutritional therapy (INT) was adopted more frequently in MDR-O SSI patients than non-MDR-O patients, but without a significant difference in duration. Patients affected by MDR-Os infections were admitted to ICU more frequently, and for a longer time than non-MDR-O SSI patients. The total postoperative course duration and the hospital stay were significantly longer in the MDR-Os SSI patients. By the multivariate regression analysis only a significant implementation of artificial nutrition, in particular INT and TPN, was evident in MDR-O-SSI patients.

**Table 4:** Postoperative course

Therapeutic measures	MDR-O SSI (*N*: 47 [%])	Non MDR-O SSI (*N*: 47 [%])	Mult. analysis OR (CI 95%)	Sig
Open abdomen	5 (10.6%)	0	–	0.3
Prosthesis	4 (8.5%)	2 (4.2%)	–	0.153
Vacuum therapy	7 (14.8%)	4 (8.5%)	–	–
MAV > 48 h	20 (42.5%)	2 (4.2%)	–	–
Tracheostomy	3 (6.3%)	0	–	0.347
Transfusions (rate)	31 (65.9%)^§§,**^	14 (29.7%)	–	0.34
TPN (%)	32 (68.8%)^§§,**^	11 (23.4%)	3.86 (1.42; 10.47)	0.008
TPN duration (days)	32.9 ± 39.9^§§,**^	9.18 ± 2.75		
INT (%)	21 (44.6%)^§§,**^	2 (4.25%)	10.02 (2.03; 49.4)	0.005
INT duration (days)	24 ± 22.2	20 ± 14.14		

*MAV* mechanical assisted ventilation, *TPN* total parenteral nutrition, *INT* immune-nutrition therapy

*p < 0.05 vs No SSI. **p < 0.01 vs No SSI. ^§^*p* < 0.05 vs non-MDR-O SSI. ^§§^*p* < 0.01 vs non-MDR-O SSI

The mean cost of treatment for patients without SSI was 8965 ± 6611 euros (total cost 421,382 euros), 12,202 ± 3533 euros (total cost 573,499 euros) in the presence of SSI (*p* < 0.05), and 22,473 ± 19,749 (total cost 1,056,275 euros) when the patient developed an MDR-Os SSI (*p* < 0.01). The cost of the antibiotic therapy, based on the AIFA prices in Italy, was 300 euros for the group of patients without SSI, 9700 euros for the group with SSI, and 115,000 euros for the group affected by MDR-O SSI.

### Microbiology

**Fig. 1:**
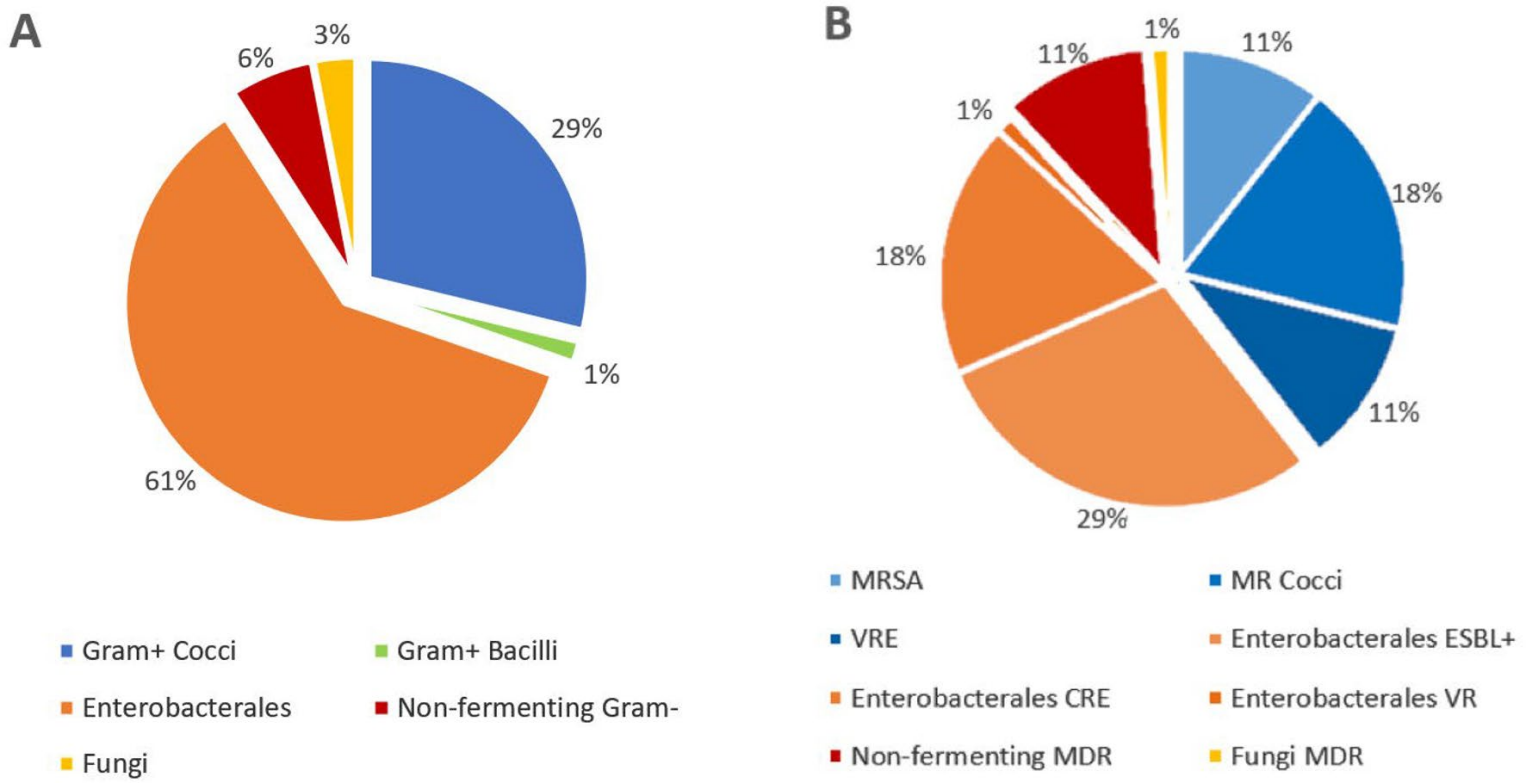
Microbiology of non-MDR-Os (**A**) and MDR-Os (**B**) cultures. **A** The first graph represents the class distribution of non-MDR pathogens in our sample. Gram+ Cocci: *S. aureus* (6.06%), *S. epidermidis* (1.51%), *S. parasanguinis* (1.51%), *S. salivaris* (1.51%), *E. faecalis* (7.57%), *E. faecium* (9.09%), *E. casseliflavus* (1.51%). Gram + Bacilli: *B. cereus* (1.51%). Enterobacterales: *E. coli* (36.36%), *Klebsiella* spp. (9,08%), *E. cloacae* (3.03%), *P. mirabilis* (1.51%), *S. marcescens* (3.03%), *M. morganii* (7.57%). Non-fermenting Gram−: *A. baumannii* (6.06%). Fungi: *C. albicans* (3.03%). **B** The second one represents the distribution of MDR pathogens based on the type of antimicrobial resistance. Gram+ Cocci (blue shades): MRSA (10.52%); MR Cocci with *S. hominis* (3.94%), *S. haemolyticus* (9.21%), *S. epidermidis* (2.63%), *S. capitis* (1.31%), *S. mitis* (1.31%); VRE with *E. faecalis* (2.63%), *E. faecium* (7.89%). Enterobacterale (orange shades): ESBL + with *E. coli* (21.05%), *K. pneumoniae* (7.89%); CRE with *E. coli* (3.94%), *K. pneumoniae* (14.47%); VRE with *E. coli* (1.31%). Non-fermenting Gram−: A*. baumannii* (2.63%), *P. aeruginosa* (7.89%). Fungi: *C. glabrata* (1.31%). *MRSA* methycillin-resistant *Staphylococcus aureus*; *VRE* vancomycin-resistant Enterococci, *ESBL+* extended spectrum beta-lactamase

Patients affected by non-MDR-Os SSI had more Gram negative *Enterobacteriales* (61%) than Gram positive (30.5%) cultures. *Escherichia coli* (36.3%) was the most frequently isolated bacterium, followed by *Enterococcus faecium* (9.09%), *Morganella morganii* (7.58%), *Staphylococcus aureus* (6%) and *Pseudomonas aeruginosa* (6%). Yeastes (*Candida albicans*) were isolated in two cases (3%) (Fig. 1a). In patients with MDR-Os SSI, *Staphylococcus species* (MRSA) were isolated in 21 (27.6%) cases, *Escherichia coli* (ESBL, CRE) in 20 (26.1%), *Klebsiella pneumoniae* (ESBL, CRE)in 17 (22%), and *Pseudomonas aeruginosa* (MDR) in 6 (7.89). *Enterococcus* species (VRE), *Acinetobacter baumani* (MDR) and *Candida glabrata* (MDR) were isolated in a limited number of cases (Fig. 1b). Extended spectrum β-Lactamase resistance was the most frequent type (31.5%), followed by methicillin resistance (27.6%), carbapenem resistance (18.4%) and vancomycin resistance *Enterococcus* sp. (11.8%). Patients affected by MDR-Os were 43 (91%), by XDR-Os were 3 (6.3%), and only 1 was PDR-Os.

Multi-bacterial infections were more frequent in MDR-Os versus non-MDR-OS patients (38.3% versus 14.8%, *p* < 0.01), were found at the first culture of biologic sample in four cases (22.2%), and emerged in 14 cases during antibiotic therapy, as effect of the antibiotic pressure on the bacterial *resistoma*. The association between multi-drug resistance and multi-bacterial infection was the most predictive factor of major surgical complications: 12/18 (66%) patients with this characteristic had grade 4 and 5 Clavien–Dindo complications.

### Discussion

This is a prospective, case–control study, focused on the treatment of MDR SSI in general surgery.

The main drawback of the study is that it is a single center study and it comes from a large University Hospital of a highly industrialized urban area. However, MDROs-SSI are probably under-reported by general surgeons, since nearly 1/3 of the SSI infections in our series were related to MDR-Os, a very high incidence that should be considered with alarm.

Resistance to the antibiotics is a typical characteristic of bacteria. It might be pre-existent and activated by antibiotics, transferred from resistant to nonresistant bacteria as long as the infection and its causes persist, and enhanced by the suppression of sensible with relative expansion resistant strains. Thus, the pathways of MDR-Os infection are multifactorial and several clinical conditions are able to trigger these effects. Previous administration of immunosuppressive agents [32], antibiotics, and hospital admission [8, 9, 19] are reported risk factors for MDR-Os expansion. However, in our series other known factors for SSI were associated to MDR-O-SSI: emergency operation [33], presence of abscess and dirty operations [34], oncologic surgery [15, 16], IBD surgery [35, 36], and visceral anastomosis [13]. An elevated ASA score, obesity, and malnutrition (low levels or plasma proteins, albumin and transthyretin) were also associated to MDR-Os infection [37, 38]. Furthermore, the degree of MDR-Os infection was all the higher, the longer was the antibiotic treatment used to overcome the original infection: in our series only 8.5% of the MDR-Os-SSI infections was poly-microbial at presentation, but a further 29.7% became MDR-Os poly-microbial during the clinical course. The development of new MDR-Os was related to the length of the antibiotic treatment and the number of the antibiotics used. However, extended or pan-drug resistance were rare and did not cause a worsening of the prognosis, as reported in the field of hepato-biliary, lung and hepatic transplantation surgery [17–19].

Only culture-guided use of antibiotics can reduce inappropriate prescription and the risk of MDR-Os development [39–42], but a further approach could be to recognize patients at risk of MDR-Os before culture, to reduce risk factors. Unfortunately, risk factors for MDR and non-MDR infections are similar, and their correction often requires a longer hospital stay which in turn is another risk factor for MDR infections (e.g. as it is in our series for low transthyretin levels associated to MDR-Os-SSI in multivariate analysis).

Looking to surgical factors associated to MDRO-S-SSI, it is always difficult to distinguish between causes and effects of the infection. We know that the risk of SSI is higher after dirty operations [34], in emergency [33] and after open laparotomy [24, 43]. In selecting patients for the control group, we have offset these factors and found that MDR-Os-SSI was associated to major surgical complications (according to Clavien–Dindo class III–V), suture dehiscence and organ-space SSI, with a significantly higher use of resources and reoperation rate. The microbiology of infected patients demonstrated a high frequency of Gram-positive *Cocci* (39 vs 29%) and a low frequency of Gram negative *Enterobacteriales* (48.6 vs 61%) in MDR-Os SSI in comparison to non-MDR-Os SSI. Extended spectrum β-Lactamase resistance was found more frequently (31.5%) than methicillin resistance in *Staphylococcus* sp. (27.6%), Carbapenem resistance of *Enterobacteriales* (18.4%) and vancomycin resistance of *Enterococcus* sp. In the first decade of twenty-first century, MRSA *Staphylococcus aureus* was the most diffuse multi-drug resistant bacteria [42], but in the last 10 years, according to our experience, there were several observations [15, 16, 18–21, 32] of MDR-Os SSI caused by Gram negative bacteria, bearing ESBL, VRE and CRE resistance. According to the classification of Majiorakos [10], the majority of antibiotic-resistant bacteria in our series were MDR (90%), and only 10% were XDR or PDR (one patient), but this microbiologic profile could be sufficient to reduce the efficacy of standard short-term antibiotic prophylaxis [14], and of empiric interval therapy [21] started before the results of microbiologic cultures. Albeit the best antibiotic therapy, the infection persists until the barrier between the external world and the “milieu interieur” is restored. To achieve this effect, we need to support the immune response, to correct malnutrition, and to avoid dehiscence by the use of ostomies in a septic condition. Takahashi et al. [13] demonstrated that MDR-Os SSI were more frequent when hepatectomy was combined with biliary tract resection (39.1% against 15.8%), due to the risk associated to bilio-intestinal anastomosis.

Finally, the treatment of MDR-Os patients had elevated costs in terms of duration of hospitalization, antibiotic therapy, and critical care [6]. Hospitalization costs increased by 36% in non-MDR-Os SSI and by 150.6% in MDR-Os SSI patients. The increase in costs for antibiotic therapy was 0.07% in patients without SSI, 1.59% in non-MDR-Os SSI, and 10.88% in MDR-Os SSI. Costs, in this series, were calculated on the basis of the Italian National Health System reimbursement program, but they could also be considered in other countries with different programs.

In conclusion, MDR-Os SSI are increasing, seem to be associated with deep protein malnutrition, major postoperative complications, suture dehiscence and iterative surgery; they are difficult to prevent, demanding in terms of clinical workload and costs of care and need a wise surgical program.

## Abbreviations

SSI: Surgical site infections
MDR-Os: Multi-drug resistant organisms
XDR-Os: Extended-drug resistant organisms
PDR-OS: Pan-drug resistant organisms
HAI: Healthcare associated infections
CDC: Center for Disease Control
ECDC: European Center for Disease Control
CLABSI: Central line-associated bloodstream infections
CAUTI: Catheter associated urinary tract infections
VAP: Ventilator-associated pneumonia
TPN: Total parenteral nutrition
INT: Immuno-nutritional therapy
ICU: Intensive care unit
qSOFA: Quick sequential organ failure assessment score
MIC: Minimum inhibiting concentration
MRSA: Methicillin resistant *Staphylococcus aureus*
VRE: Vancomycin resistant *Enterococcus faecium*
ESBL: Extended spectrum beta-lactamase
CRE: Carbapenem resistant Enterobacteriales
IBD: Inflammatory bowel diseases
MAV: Mechanical assisted ventilation
